# Notes for Contributors

**DOI:** 10.1155/S1463924679000663

**Published:** 1979


### Computing integrator

Pye Unicam have reached an agreement with Spectra Physics to market their SP4100 computing integrator in the UK. The SP4100 is fully compatible with Pye Unicam's range of Gas and Liquid Chromatographs. It is microprocessor controlled, has a full Alphanumeric keyboard and LED display and an integral printer plotter. BASIC is standard in the SP4100 with extensive built-in programming which can be implemented with just a few key strokes.

Built in BASIC programming capability allows the user flexibility to modify an existing data reduction method, format his own report or develop other custom applications. The user can modify, through a programme overlay, the BASIC preprogrammed methods in ROM.

The plotter prints at 24 characters per second in black on white and can 'chart reverse', thus enabling it to do X-Y plotting such as graphing a multi level calibration. It has a full Alpha Numeric keyboard with both upper and lower case characters plus 15 function keys labelled in familiar chromatography terms. There is an intelligent interactive dialogue and automatic dynamically optimised integration parameters.

*Pye Unicam Ltd, York Street, Cambridge CB1 2PX, UK.*

### Sample concentrator

The evaporation of solvents from samples can be a time consuming operation in the preparation of samples for analysis. The Techne SC-3 sample concentrator can be used for any multi-evaporation requirement of solvents in test tubes or centrifuge tubes. It is particularly useful when using thermolabile substances where it is possible to effect adequate evaporation rates at lower temperatures than with heating alone. Any size of standard test tube in the range of 6 mm to 25 mm can be used with the unit which can handle up to 90 of the 6 mm size tubes. Gas, $CO_2$ or $N_2$ is introduced into the test tube by a multi-outlet gas reservoir via hypodermic needles.

*Techne Cambridge Ltd, Duxford, Cambridge, CB2 4PZ, UK.*

### UV Detector

Schoeffel Instrument Division of Kratos have recently introduced an ultraviolet detector for HPLC. The detector, the model SF 740, employs a deuterium lamp and can therefore perform absorption measurements at any wavelength between 200 and 380 nm without changing the lamps. Quick-change filter cassettes isolate the wavelength of interest. Standard filters available include 200, 215, 240, 254 and 280 with other wavelengths available on special order. The use of shorter UV wavelengths has been found useful in the detection of amino acids, peptides, many carbohydrates, and many other compounds.

The detector features an easily removed and cleaned flowcell with a small 10 $\mu l$ volume. Nine calibrated absorbance ranges from 0.005 to 2.0 AUFS are push-button selectable. Noise in this single beam reference compensated detector is less than $1 \times 10^{-4}$ AU. The SF 740 can be interfaced with any liquid chromatography.

*Kratos Inc, Schoeffel Instrument Division, 24 Booker Street, Westwood, NJ 07675, USA.*

### Blood chemistry analyser

An automatic blood chemistry analyser, the Hitachi Abca, was displayed for the first time in the UK at the International Clinical Chemistry Congress in Brighton by LKB. The analyser is capable of selective profiles of up to twelve chemistries but follows the philosophy of discrete analysis. It embraces a reaction cup laundry system and is simple to operate because of its microprocessor control and reporting system.

*LKB Clinicon Systems Ltd, Bell Lane, Lewes, East Sussex BN7 1LG, UK.*

### Notes for Contributors

### Presentation of manuscripts

Manuscripts should be typed (double-spaced) on one side of the paper only and with generous margins. The title should be brief and informative avoiding the word "new" and its synonyms. The full list of authors with their affiliations and full address(es) should appear on the title page. On a separate sheet an abstract of no more than 150 words is required. This should succinctly describe the scope of the contribution and highlight significant findings or innovations. It should be written in a style which can easily be translated into French and German.

*The Concise Oxford Dictionary* and Fowler's *Modern English Usage* (both published by Oxford University Press) should be used as the standard for spelling and grammar. Abbreviations should be limited to those generally recognised, or where a frequently occuring term is abbreviated it should, in the first instance, be explained thus "flow injection analysis (FIA) ..." and the abbreviation used thereafter. Abbreviations, for standard measures and units should follow SI recommendations. There are various publications giving guidance on the use of SI units.

References should be indicated in the text by numerals following the author's name, i.e. Skeggs [6]. On a separate sheet of paper, list all references in numerical order thus:

[6] Skeggs, L.T., *American Journal of Clinical Pathology,* 1959, **28**, 311.

Note that journal titles are given in full. Where there is more than one author, the form Foreman et al. should be used in the text but all authors should be named in the list of references. When reference is made to a chapter in a book the reference should take the following form:

[7] Malmstadt, H.V. in "Topics in Automatic Chemistry" Ed. Stockwell P.B. and Foreman J.K. 1978 Horwood, Chichester, pp. 68-70.

Only work which has been published or has been accepted for publication should be cited. Avoid the citation of documents which are subject to restricted circulation, patent literature, unpublished work and personal communications. The latter can be mentioned in the text in parenthesis.

To illustrate a paper line diagrams are preferred to photographs. Photographs should only be used when they significantly add to the discussion. Diagrams, charts and graphs should be carefully drawn in black ink on stout card or heavy quality tracing paper. Most illustrations are reduced for publication; to allow for this originals should be between 16 and 36 cm wide (the depth must not exceed 50 cm). The lettering of diagrams should be sufficiently clear to withstand reduction. Except in the case of proper names, all lettering should be in lower case print. If photographs are used they must be supplied in the form of clear, unmounted, glossy, black and white prints. "Instant" photographs are not normally acceptable. All illustrations must be identified on the reverse showing the figure number and the author's name.

Each illustration should have a fully explanitory caption. Captions should be typed together on a separate sheet of paper; they must *not* be an inseparable part of the illustration.

### Amino acid analyser

LKB has announced the availability of their 4400 Amino acid analyser. The instrument provides fast analysis times for both protein hydrolysates (65 minutes) and physiological fluid. This performance is achieved using a horizontal, stainless steel analytical column mounted in a solid-state heating system and a high temperature reaction coil for fast colour development. It has a 70 sample, cooled autoloader and optical data-handling accessory.
*LKB Instruments Ltd, 232 Addington Road, Selsdon, South Croydon, Surrey, CR2 8YD, UK.*

### Automatic clinical analyser

An automatic clinical analyser has been developed by Du Pont. The analyser, the 'aca' III, currently under evaluation in Europe, incorporates the same characteristics as the present 'aca' instruments. It includes these additional features: computer assisted method calibration, automatic instrument standardisation, instrument diagnostic programs, expanded report format and alphanumeric keyboard and display. It has the capacity to allow new methods to be added as they are developed. Vital parameters are displayed in a selected language to aid instrument operation. The instrument is compatible with any on-line laboratory information system.
*Du Pont de Nemours Belgium SA, Instruments Products Division, Mecure Center, 1130 Brussels, Belgium.*

### Spectrophotometer

Du Pont's UV spectrophotometer, introduced at the Pittsburgh Conference on Analytical Chemistry, is a compact variable wavelength detector. The modular detector allows selection of the optimum operating wavelength specific to the absorption of the compound to be measured. When operated at the UV absorption maximum of the compound being measured, sample detection sensitivity can be increased.

The spectrophotometer incorporates a stable air-cooled deuterium source and a high efficiency dual beam grating monochrometer. Absorbance ranges are adjustable from 0.005 to 2.56 AUFS in ten steps. The unit complements their 850 and 860 HPLC systems and it is compatible with most liquid chromatographs.
*Du Pont (UK) Ltd, 64a Wilbury Way, Hitchin, Herts SG4 0UR, UK.*

### Automatic melting points

An automatic melting point instrument, the Mettler FP61, is available from MSE Scientific Instruments. It is portable and simple to use. The melting point is indicated on a digital display readable to 0.1 °C and the melting process can be graphically reproduced by a recorder connected to the instrument.
*MSE Scientific Instruments, Manor Royal, Crawley, Sussex, UK.*

### Calendar

**Editor's Note:**
***Organisers of conferences, seminars etc. should send details for inclusion in this calandar as soon as the relevant information is available and not later than three months before the event.***

**Synthesis in Organic Chemistry.**
July 23-26, Cambridge, UK.
*Dr. M. Robinson, The Chemical Society, Burlington House, London W1, UK.*

**3rd International Conference on the Chemistry and Uses of Molybdenum.**
August 19 - 23, Michigan
*Climax Molybdenum Co. Ltd., 1 - 3 Grosvenor Place, London SW1, UK.*

**1st European Symposium on Organic Chemistry.**
August 20 - 23, Cologne, W. Germany
*Dr. W. Fritsche, c/o Gesellschaft Deutscher Chemiker, PO Box 90 04 40, D-6000 Frankfurt, Main 90, FRG.*

**5th Australian Symposium on Analytical Chemistry.**
August 20 - 24, Perth, Australia
*B. Codling, c/o Government Chemical Laboratories, 30 Plain Street, Perth, WA 6000, Australia.*

**Automatic methods in connection with Trace-organic Analysis**
September 4 - 7, Guildford, U.K.
*Dr. E. Reid, Wolfson Bioanalytical Centre, University of Surrey, Guildford GU2 5XH, U.K.*

**Automation and Computerisation in the Medical Laboratory**
September 5 - 7, Stirling University, Scotland.
*G.W. Thomson, Secretary, IMLS Glasgow Branch, 67 Glen Oglivie, St. Leonards, East Kilbride, Scotland.*

**Management Studies for R & D Chemists.**
September 10 - 14, Slough, UK.
*Mrs. S. Leclercq, The Chemical Society, Burlington House, London W1V 0BN, UK.*

**Laboratory 79**
September 11-13, London, UK.
*Curtis Steadman, 26-34 High Street, Saffron Waldon, Essex CB10 1EP, UK.*

**International Conference on Flow Analysis**
September 11 - 13, Amsterdam, The Netherlands.
*Secretary FA-Amsterdam,. Laboratory for Analytical Chemistry, University of Amsterdam, Nieuwe Achtergracht 166, 1018 WV Amsterdam, The Netherlands.*

**Communications in Microprocessor Industrial Instrumentation**
September 12 - 13, London U.K.
*Mrs. P. Keiller, Sira Institute, South Hill, Chiselhurst, Kent BR7 5EH, U.K.*

**Ninth International Meeting on Organic Geochemistry**
September 17 - 20, Newcastle-upon-Tyne, U.K.
*Dr.A.G. Douglas, Organic Geochemistry Unit, Geology Department, Drummond Building, The University, Newcastle-upon-Tyne, NE1 7RU, U.K.*

**Colloquium: Apport de l'informatique a l'analyse industrielle pour le controle et la conduite des procedes.**
September 18 - 19, Lyon
*Societe de Chimie Industrielle, 28, rue Saint-Dominique, 75007 Paris, France.*

**Automatic on-line industrial chromatograph: sophisticated analyser or industrial transducer? – colloquium.**
October 3 - 5, Arles
*Le Comite d'Organisation, Institut de regulation et automation, Chemin des Moines, 13644 Arles, France.*

**Real-time Datahandling and Process Control**
October 23 - 25, Berlin, West Germany
*Congress Organisation Company, John Foster Dulles Allee 10, D-1000 Berlin, West Germany.*

**International Liquid Chromatography Symposium IV.**
October 24 - 25, Strasbourg, France.
*X Delemere, Waters Associates SA, 18 rue Goubet, 75019 Paris, France.*

**1980**

**Automation at SAC 80**
July 20 - 26, Lancaster, U.K.
*The Secretary, Analytical Division, The Chemical Society, Burlington House, London, W1Y 0BN, U.K.*

**1981**

**Euroanalysis IV**
August 23 - 28, Helsinki, Finland.
*Association of Finnish Chemical Societies. Executive Secretary, Pohj, Hesperiankatu 3B10, SF-00260 Helsinki 26, Finland.*

